# A Metabolism Toolbox for CAR T Therapy

**DOI:** 10.3389/fonc.2019.00322

**Published:** 2019-04-30

**Authors:** Xuequn Xu, J. N. Rashida Gnanaprakasam, John Sherman, Ruoning Wang

**Affiliations:** Center for Childhood Cancer and Blood Diseases, Hematology/Oncology & BMT, The Research Institute at Nationwide Children's Hospital, Ohio State University, Columbus, OH, United States

**Keywords:** immunotherapy, metabolism, chimeric antigen receptor (CAR), tumor microenvironment (TME), anti-tumor immune response

## Abstract

The adoptive transfer of T cells expressing chimeric antigen receptors (CARs) through genetic engineering is one of the most promising new therapies for treating cancer patients. A robust CAR T cell-mediated anti-tumor response requires the coordination of nutrient and energy supplies with CAR T cell expansion and function. However, the high metabolic demands of tumor cells compromise the function of CAR T cells by competing for nutrients within the tumor microenvironment (TME). To substantially improve clinical outcomes of CAR T immunotherapy while treating solid tumors, it is essential to metabolically prepare CAR T cells to overcome the metabolic barriers imposed by the TME. In this review, we discuss a potential metabolism toolbox to improve the metabolic fitness of CAR T cells and maximize the efficacy of CAR T therapy.

## Cancer Cell Metabolic Program

Since cancer cells must constantly proliferate, they must also continuously generate new biomass. This in turn requires a substantially different metabolic program than that of non-proliferating somatic cells. Most non-proliferating cells utilize oxidative phosphorylation (OXPHOS) to efficiently extract ATP from pyruvate, while cancer cells reduce the majority pyruvate into lactate, a process termed aerobic glycolysis or “the Warburg Effect” (1). In 1956, Otto Warburg observed the tendency of cancer cells to metabolize glucose into lactate instead of carbon dioxide and concluded that cancer was a disease of damaged respiration (2). While not all of Warburg's conclusions have stood the test of time, it holds true that metabolism is a critical component of oncogenesis. Even if a cell has developed mutations to overcome the normal regulation of proliferation, it also requires a metabolic program that will allow the cell to synthesize all the molecules required for a new cell. Metabolism is so critical to oncogenesis that the most commonly mutated pathways, including Ras, Phosphoinositide 3-kinases (PI3K)/AKT/mammalian target of rapamycin (mTORC1), hypoxia inducible factor 1 (HIF-1), proto-oncogene MYC (c-MYC), and p53, are key metabolic regulators. HIF-1 and c-MYC in particular act in concert to express glucose and lactate transport proteins while diverting pyruvate away from OXPHOS and toward lactate production (3–5). While this method does not maximize the amount of ATP that can be extracted from glucose, it is none the less advantageous for proliferating cells. By keeping glucose derived carbon out of the TCA cycle, additional carbons are made available for lipid, protein and especially nucleotide synthesis (1, 6, 7). Sequential activation of PI3K, followed by Akt and mTORC1, are also able to aid cancer cells in capturing glucose from the environment, as well as catabolic metabolites from the mitochondria. Akt activates hexokinase and phophofructokinase-1, to retain glucose and commit it to further glycolysis, as well as ATP-citrate lyase, to convert mitochondrial citrate into cytosolic acetyl-CoA for lipid synthesis (7, 8). Finally, mTORC1 enhances mitochondrial biosynthesis to take oxaloacetate and α-ketoglutarate (α-KG) and convert them into amino acids for protein synthesis (7). Alternative sources of carbon can support growth in cell size, but glucose is required for robust DNA synthesis and concomitant cell cycle progression (9). The other major carbon and energy source is glutamine. Upon being metabolized by the cell, glutamine is shuttled into the TCA cycle to make α-KG for the production of amino acids, or citrate for the production of lipids (1, 6). Loss of p53 additionally allows malate to leave the TCA cycle to be converted into pyruvate, generating additional NADPH, another necessity for nucleotide synthesis (10). Reduction of pyruvate into lactate serves to regenerate NAD^+^ for further glycolysis while also conditioning the extracellular space (11).

The disorganized structure of a solid tumor means that it is not properly enervated by blood vessels. Cells located farther from the blood vessels experience hypoxia, nutrient deprivation and acidosis (3, 12). Cancer cells experiencing nutrient deprivation must rely on alternative metabolites or methods of nutrient acquisition. Ras expressing cancer cells may use macropinocytosis to scavenge nutrients from the surrounding milieu. Further, Ras driven cells utilize autophagy to degrade unnecessary cellular components into small molecule nutrients (12). Alternatively, hypoxia re-enforces glycolytic generation of lactate, while also shunting glutamine toward lipid genesis (3, 12). Finally, lactate is associated with several oncogenic processes, such as angiogenesis, cell migration and immune suppression (5). Not strictly a waste product, cells that experience chronic acidosis may take up lactate for gluconeogenesis and use in oxidative phosphorylation (11). Thus, as a tumor progresses, cancer cells condition the microenvironment, creating unique selection pressures and contributing to further heterogeneity and metabolic derangement.

## T Cell Metabolic Program

T cells play a key role in mounting a robust, antigen specific adaptive immunity against invading pathogens and tumor. Upon stimulation of antigen receptors, naïve T (T_n_) cells rapidly transit from a quiescent to an active state that begins with a 24 h growth phase followed by massive proliferation, differentiation, and migration. To elicit a robust immune response, T cells can differentiate into diverse functional subsets. Depending on the cytokine milieu of the microenvironment, active CD4^+^ T cells can differentiate into immune suppressive regulatory T (T_reg_) cells or inflammatory T effector cells, such as T helper T_H_1, T_H_2, T_H_9, T_H_17 and follicular helper T (T_fh_). On the other hand, active CD8^+^ T cells mainly differentiate into CD8^+^ effector T (T_eff_) cells, also referred as cytotoxic T lymphocytes (CTL). Following pathogen clearance, the majority of the effector cells die through apoptosis while the remaining cells survive to form a population of long-lived memory T (T_mem_) cells, responsible for immunity upon subsequent challenges to the same pathogen. T_mem_ cells are composed of distinct subsets including stem memory cells (T_scm_), central memory cells (T_cm_), and effector memory cells (T_em_). T_scm_ cells exhibit a naïve like phenotype (CD44^−^CD62L^+^/CD45RA^+^), express interleukin-2 receptor (IL-2R) β and the chemokine C-X-C motif receptor 3 (CXCR3), representing the earliest and long-lasting developmental stage of T_mem_ cells. T_scm_ cells have a capacity to self-renew and generate the entire spectrum of more differentiated cells. T_cm_ cells are CD62L^+^, reside in lymph nodes and have limited or no effector function, but they proliferate and become effector cells upon secondary stimulation. These cells represent an intermediate population between T_scm_ cells and T_em_ cells. T_em_ cells are CD62L^−^, are the progenitor cells prone to differentiate into T_eff_ cells upon secondary stimulation. Therefore, T_em_ are responsible for protective memory, and migrate into inflammatory tissues to elicit an immediate response against antigens (13).

T cell activation and differentiation are accompanied by dramatic shifts in cellular metabolic programs which fulfill their bioenergetic, biosynthetic and redox demands (14–19). Essentially, different phenotypic and functional T cell subsets are characterized by unique metabolic demands, which are tightly linked with immune modulatory signaling cascades. Specifically, quiescent T_n_ and T_mem_ cells rely on fatty acid oxidation (FAO) and OXPHOS to maintain their basic energy level, cellular function and viability (20, 21). In addition, heightened glycerol uptake and triglyceride synthesis also play an important role in promoting memory CD8^+^ T cells (22, 23). Overall, active T cells predominantly engage in aerobic glycolysis, the pentose phosphate pathway (PPP) and glutaminolysis to drive proliferation and subsequent effector functions (20, 24–28). However, it remains unclear whether a shift in glucose metabolism promotes activated T cells to become long-lived T_mem_ cells. It has recently been suggested that persistently heightened glycolysis in T_eff_ cells compromises the formation of long-lived memory cells by driving T cells toward a terminally differentiated state, resulting in a failure to survive upon adoptive cell transfer, whereas a moderately dampened glycolysis supports the generation of long-lived memory CD8^+^ T cells and enhances anti-tumor immune response (29, 30). CD4^+^ effector T cells including T_H_1, T_H_2, T_H_9, T_H_17, and T_fh_ cells display heightened glycolysis, whereas FAO activity supports the differentiation and function of T_reg_ cells (31–37). Conversely, heightened aerobic glycolysis is eventually required to drive T_eff_ cells proliferation and differentiation into cytotoxic T cells (38).

Catabolism of glucose and glutamine generates energy, provides reducing power, and donates carbon and nitrogen to biosynthetic building blocks. During the sequential reactions of aerobic glycolysis, the breakdown of glucose into lactate generates ATP and intermediate metabolites, many of which are channeled into the biosynthesis of amino acids, lipids and nucleotide. Branching from glycolysis, the PPP starts from glucose-6-phosphate, an immediate metabolic product of glucose, and produces NADPH in the oxidative phase as well as five-carbon sugars in non-oxidative phase, the latter of which can feed back into glycolysis or provide precursors for nucleotides. Meanwhile, NADPH is required for modulating redox balance and cholesterol biosynthesis (17, 20, 39). During glutaminolysis, glutamine is converted to glutamate and subsequently to α-KG, which serves as an important anaplerotic substrate of the TCA cycle and fuels mitochondrial ATP production. As a major source of carbon and nitrogen, the catabolic products of glutamine are funneled to support the biosynthesis of amino acids, hexosamine, polyamine, lipids and nucleotides during T cell proliferation and differentiation (40–43). Similar to glucose catabolism, glutamine catabolism is composed of multiple metabolic routes branched from glutaminolysis. Glutamate is the key branch point in glutaminolysis and can be committed toward the biosynthesis of glutathione (GSH) or toward mitochondrial oxidation to produce biosynthetic precursors and ATP. Glutamate is derived from glutamine in parallel through glutaminase, in mitochondria, as well as through glutamine utilizing enzymes, including amidotransferase, largely in cytosol (44–47). As such, the subcellular compartmentalization of glutamate may represent an important mechanism that enables a fine-tuned coordination between branched metabolic routes to fulfill bioenergetics, biosynthetic and redox demand in T cells (36, 48, 49).

T cell metabolic reprogramming during activation is strictly regulated by numerous kinase signaling cascades, including mitogen-activated protein kinase (MAPK)/extra-cellular signal-regulated kinase (ERK), mTOR kinase, AMP-activated protein kinase (AMPK), and PI3K/Akt (20, 24, 50–55). T cell activation requires co stimulation of CD28 and IL-2 signaling, which activates PI3K/AKT and mTOR to promote the uptake of glucose and amino acids to support CD4 T cell proliferation and differentiation (31, 56). AMPK and mTOR coordinate metabolic status with signaling transduction in regulating T cell differentiation. T_H_1, T_H_2, and T_H_17 subsets, which predominantly rely on glycolysis, maintain high mTOR activity. Conversely, T_reg_ cells, which require FAO, maintain high AMPK activity. In addition, inhibition of mTOR complex 1 (mTORC1) by rapamycin or Wnt-β-catenin signaling in T cells drives the differentiation of T_scm_ cells by switching T cell metabolism toward FAO and increasing the long term survival (57, 58).

Beyond these key kinase-signaling cascades, (HIF-1) and the (c-Myc), are the key transcription factors that regulate the expression of metabolic enzymes in glucose and glutamine catabolism (20, 32, 59, 60). In addition, activating enhancer binding protein 4 (AP4), Activating transcription factor 4 (ATF4), interferon regulatory factor 4 (IRF4), Bcl-6 are involved in regulating the expression of various metabolic genes to promote glycolysis and glutaminolysis as well as T_eff_ function (61–63). On the other hand, *de novo* cholesterol biosynthesis is regulated by the dynamic regulation of nuclear receptor- liver X Receptor (LXR) Foxo1 protein (Foxo1) and the orphan steroid receptor, Estrogen-related receptor alpha (ERRα) (31, 33, 40, 64, 65).

## Metabolic Antagonism in the TME

Emerging evidence suggests that various metabolites from various cellular compartments within the TME may serve as a complex form of intercellular communication which modulates tumor cell growth and response to therapy (66–72). T cell metabolic pathways are tightly and ubiquitously linked with T cell activation, proliferation, differentiation, and immune functions (24, 25, 27, 31, 39, 39, 51, 56, 73). Thus, the immune cells, particularly effector T cells, are intimately controlled by the metabolic communications in the TME.

### Nutrients Depletion

In addition to lineage-specific metabolic requirements, which are associated with the metabolic network in the tissue-of-origin, cancer cells display a heightened ability to capture carbon and nitrogen sources from the TME and process these raw materials to meet the cell's fundamental requirements for energy, reducing power and starting materials for biosynthesis. These general metabolic features of cancer cells are required to support the needs imposed by proliferation and other neoplastic features, but at the same time often deplete the TME of nutrients (74, 75). In addition to the consumption of key carbon and nitrogen sources, glucose and glutamine, rapidly proliferating cancer cells and T effector cells have a strong demand for amino acids, some of which are not only required for protein synthesis, but are also coupled to other anabolic routes and therefore integrated into central carbon metabolism. However, both cancer and T effector cells are often dependent on the uptake of extracellular substrates from the TME, as opposed to *de novo* biosynthetic pathways, which are either defective or insufficient to fulfill the demands. It is well-documented that high expression of indoleamine-2,3-dioxygenase (IDO) and tryptophan-2,3-dioxygenase (TDO) by macrophages and cancer cells contributes to immune tolerance by mediating the conversion of tryptophan to kynurenine (76–79). Tryptophan depletion and kynurenine accumulation cooperatively suppress anti-tumor immunity by reciprocally impairing the growth and survival of T effector cells and enhancing the development and function of Tregs and myeloid-derived suppressor cells (MDSC) (80–85). Extracellular cysteine and arginine are also important nutritional resources, which both T and cancer cells compete over. Cysteine, alone with glycine and glutamate, are the substrates for the *de novo* synthesis of GSH, which is the most abundant cellular antioxidant, to ensure physiological levels of intracellular reactive oxygen species (ROS) (20, 36, 48, 49, 51, 73, 86, 87), While glucose and glutamine catabolism provide glycine, glutamate and reducing power though NADPH, proliferating cells largely obtain cysteine from the local microenvironment (20, 86, 88–101). Lack of cystathionase, the enzyme that converts methionine to cysteine, may render T cells particularly vulnerable to cysteine starvation compared to cancer cells (102). Supplementing T cells with arginine has been shown to promote the production of pro-inflammatory cytokines as well as a central memory phenotype *in vitro*, while also enhancing the T cell-mediated antitumor response *in vivo* (103–107). Conversely, the production of the arginine-degrading enzyme, arginase, in the TME has been known to causes arginine depletion and T cell anergy (104). Further, nitric oxide (NO), which is produced from arginine by nitric oxide synthases (NOS), may have cytotoxic effects on proliferating cells in the TME. However, mutated p53 may confer the cancer cells with enhanced resistance to NO-mediated cytotoxicity when compared to T effector cells (108–111).

### Accumulation of Immune Suppressive Metabolic End-Products and By-Products

A fierce competition for limited carbon and nitrogen sources between tumor and T effector cells leads to the depletion of nutrients and accumulation of metabolic end-products and by-products, the latter of which also has a profound impact on T effector cells. Accumulation of lactic acid and CO_2_ results in the acidification of the TME, which suppresses T cell proliferation and impairs cytokine production and cytotoxic activity of T cells, while causing tumor radio resistance and promoting tumor cell migration and invasion (112–118). The acidification of the TME also profoundly impacts the cross-membrane transport of sodium ions and amino acids, as well as the pro inflammatory function of T effector cells (117–121). Additionally, tumor-derived potassium has been shown to potentially suppress T cell function (122). Stressed or damaged cells release ATP/ADP and their catabolic product adenosine into the TME, the latter of which elicits immune suppressive effects, partially through engaging cell-surface P2 purinergic receptors-mediated signaling in T cells (123–128). CD39 and CD73 are two ectonucleotidases that are widely expressed in the plasma membrane of cancer cells and cancer stromal cells and are responsible for the conversion of ADP/ATP to AMP and AMP to adenosine. As such, CD39 and CD73 play critical roles in determining the outcome of antitumor immunity through shifting ATP-driven pro-inflammatory effects to an anti-inflammatory milieu mediated by adenosine (129–131). Adenosine deaminase (ADA) converts adenosine to inosine, terminating adenosine-mediated immune suppressive effects (132). Consistent with this finding, the genetic loss of ADA results in an accumulation of adenosine, and leads to severe combined immunodeficiency (SCID) (133, 134).

The genetic context also plays a critical role in determining the composition of immunomodulatory metabolites in the TME, which differs dramatically in tumors with or without mutations in specific metabolic enzymes (135–137). R-2-hydroxyglutarate (2HG), which is enriched in tumors with gain-of-function isocitrate dehydrogenase 1/2 mutations, suppresses T cell activation and differentiation. Intriguingly, S-2HG (the other enantiomer of 2HG) supplement greatly enhances the anti-tumor capacity of adoptively transferred T cells (138). Tumors with succinate dehydrogenase (SDH) or fumarase mutations display elevated levels of succinate and fumarate, respectively. While the accumulation of intracellular 2HG, succinate and fumarate (often referred as onco-metabolites) may drive transformation in a tumor intrinsic manner, all these metabolites may indirectly contribute to tumorigenesis through their immunomodulatory effects (139, 140). A cell permeable form of fumarate, dimethyl fumarate (DMF), is the active ingredient of BG-12/TECFIDERA and FUMADERM, which have been widely used for treating autoimmune disorders (141–143). The anti-inflammatory activity of DMF has been partially attributed to its effect on suppressing T effector functions (36, 144, 145).

**Figure 1:**
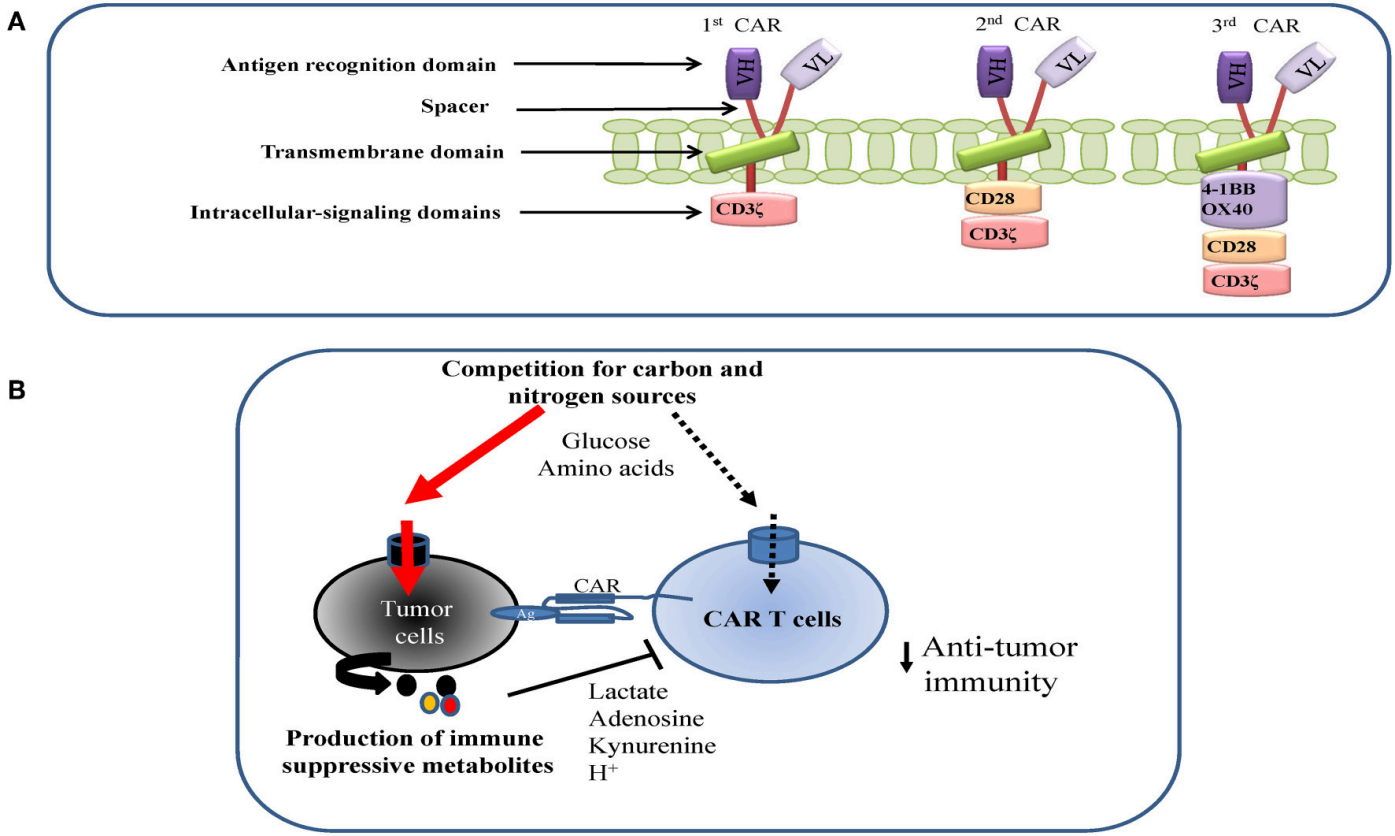
**(A)** CART design and structure. **(B)** Metabolic antagonism between tumor and CAR T cells in the tumor microenvironment (TME). Tumor cells may suppress CAR T cells proliferation and function by competing for key nutrients with CAR T cells and excreting immune-suppressive metabolites into the TME.

Depending on the nature of the metabolic stress imposed by the TME, cancer cells readily engage an array of signaling responses that are largely orchestrated by AMPK, mTOR or transcriptional factors, such as HIF1α, nuclear factor-like 2 (Nrf2), and general control non-derepressible 2 (GCN2). These stress responses are not only adaptive, but also cytoprotective and oncogenic, thus rendering cancer cells resistant to apoptosis and favoring the development of more aggressive, invasive and malignant phenotypes (146–150). Similarly, metabolic stresses favor the development of immune suppressive regulatory T (T_reg_) cells and tumor associated macrophages (TAM) (31, 32, 151–154). However, metabolic stresses are less tolerated in quickly proliferating T effector cells, leading to more cell death as well as less proliferation and cytokine production (14, 18, 39, 155–158). To survive, expand and exert robust anti-tumor activities in the TME, T_eff_ cells must efficiently overcome the metabolic stress caused by the depletion of nutrients and accumulation of immune suppressive metabolites (Figure 1B). Recent research suggests that, the transfer of less differentiated T_mem_ subsets results in greater expansion, persistence and anti-tumor efficacy than terminally differentiated T_eff_ cells. In particular, T_scm_ has a robust capacity for self-renewal and functional plasticity, though further differentiation into T_cm_, T_em_, and T_eff_ subsets, thus providing a persistent anti-tumor immunity. Therefore, these cells could effectively compete with cancer stem cells over time to eradicate the tumor mass (159–161). Overall, less differentiated T_mem_ cells require increased mitochondrial FAO and spare respiratory capacity (SRC) for their long term survival and persistence (25, 162, 163). Clearly, the metabolic fitness of T cells is essential for successful adoptive immunotherapy.

## Metabolic Optimization of the Clinical Manufacture and Application of CAR T Cells

The recent breakthroughs of CD19 CAR T cell therapies to cure hematologic malignancies provide an exciting promise of extending this approach to solid tumors (164, 165). However, a critical barrier to using CAR T therapy for treating solid tumors that express appropriate antigens is the tumor's hostile microenvironment. A plethora of immunosuppressive mechanisms imposed by tumor cells suppress T cell proliferation, survival and effector function (155–169). In addition to the cytokine-mediated and cell-surface receptor-mediated signals that are essential for suppressing T cell functions and responses, the TME represents a dramatic example of metabolic derangement. Insufficient tumor vascularization due to disorganized blood vessel networks leads to hypoxia, lack of nutrients, acidosis, and the accumulation of metabolic waste and free radicals (170, 171). In addition, the increased nutrient and oxygen demands of tumor cells imposed by heightened oncogenic signaling further aggravates the metabolic stresses that suppress effector T cells function (6, 7, 46, 74, 75, 155, 169, 172, 173). As such, a rational and effective CAR T immunotherapy for solid tumors needs to integrate novel strategies which improve T cell metabolic fitness to overcome metabolic stresses imposed by the TME.

**Figure 2:**
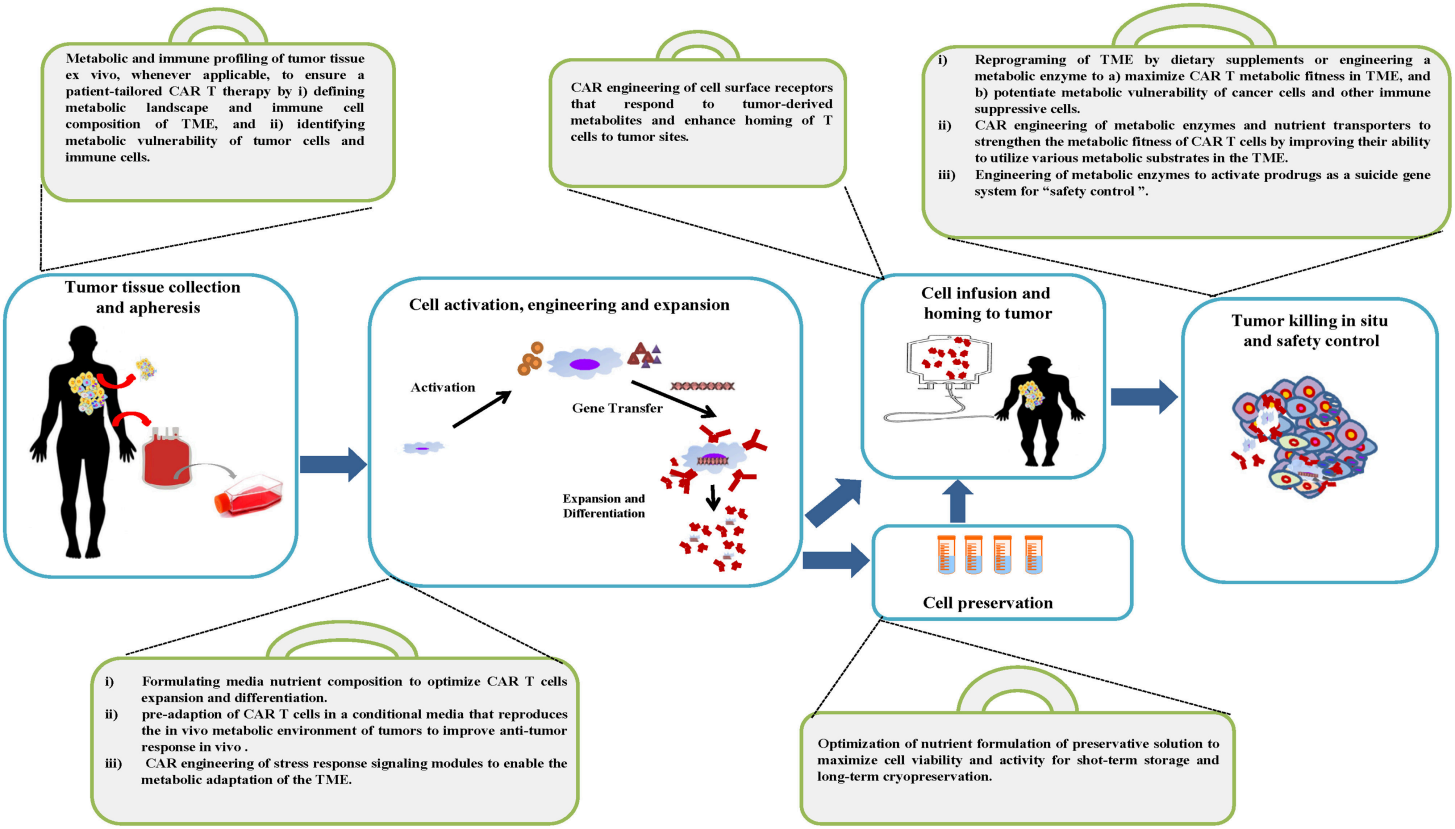
Metabolic tool box of the CAR T cell manufacturing process and therapy. A variety of metabolic strategies are proposed to be integrated into standard work flow to optimize the manufacturing process and maximize the therapeutic efficacy of CAR T cells.

The standard manufacturing process of CAR T cells starts by obtaining the patient's peripheral blood mononuclear cells (PBMC) through leukapheresis, followed by T cell enrichment, activation and gene modification with viral or non-viral methods. These genetically modified T cells are then expanded to the required cell numbers for therapy, and then formulated and/or cryopreserved before infusion into the patient (Figure 2). The production of CAR T cells requires quality control testing throughout the entire process and is subjected to Good Manufacturing Practices (GMP) guidelines (174–177). We envision using the following metabolic strategies to optimize the manufacturing process and maximize the therapeutic efficacy of CAR T cells.

### Tumor Tissue Specimen Collection Before Leukapheresis

The personalized nature of CAR T therapy requires a patient-tailored strategy to ensure the robustness and reproducibility of personalized cell products. Whenever biopsy or surgery is applicable, a step-wise system of metabolic and analytic assessment is needed to determine the *in situ* immunomodulatory metabolic landscape in human tumor tissue specimens. Recently, Stable Isotope Resolved Metabolomics (SIRM) has been applied to assess the metabolic activities of thin tumor tissue slices, an adaptation of the original method of Otto Warburg's tissue slice technique (178–180). Along with conventional untargeted metabolomics, SIRM will provide complementary information, untargeted high-resolution mapping of the metabolic fate of carbon and nitrogen atoms from labeled precursors as well as quantification of nutrients and immune modulatory metabolites. In addition, the expression profile and immune modulatory impacts can be further assessed by combining RNAseq and Metabolomics-edited Transcriptomic Analysis (META) (181, 182); Using patient-derived tissue slides in studies preserves the fidelity of the original native cellular architecture and metabolic profiling on an individual patient basis. Integration of the above proposed approaches is poised to comprehensively profile the landscape of immunomodulatory metabolism in the TME, which may facilitate complementary metabolic improvements in the following steps of the manufacturing process of CAR T cells.

### T Cell Activation, Genetic Modification, and Expansion

CARs are generated by combining the antigen-binding region of a monoclonal antibody with key stone intracellular-signaling domains. It consists of an extracellular targeting domain that recognizes a tumor specific antigen, which is derived from a single-chain variable fragment (scFv) of the variable heavy and variable light chains from a specific monoclonal antibody. When it is expressed on the surface of a T cell, the targeting domain allows CAR T cells to recognize and bind to the antigen that is presented by cancer cells. The intracellular signaling domain usually originates from the signal transduction subunit of co-stimulatory receptors, such as 4-1BB and CD28, which transduce extracellular ligand binding signal into CAR T cells to initiate the activation of downstream signaling cascades. CAR structure has been improved from first-generation CARs, which only had the CD3ζ signaling domain, to next generation CARs, which link the signaling endo domains of CD28, 4-1BB, and/or OX40 to provide co-stimulation signal (signal 2), which is required for optimal T cell activation (Figure 1A) (183–185). Optimal activation, gene transfer and culture conditions are essential to ensure the required cell number and quality are achieved for CAR T cell therapy. Due to the personalized nature of CAR T therapies, the degree of cell amplification, differentiation and functional activation can differ significantly from patient to patient. A patient tailored nutritional formulation for cell culture media may improve the robustness and reproducibility of cell expansion. In addition, emerging evidence suggests that the formula for commercial media does not truly reflect nutrient composition in a physiological context, which is required to ensure the metabolic fitness of cells *in vivo* (186–188). CAR T cells with co-stimulatory domain 4-1BB display higher mitochondrial metabolic activity than cells with a CD28 co-stimulatory domain (30). Consistent with this finding, mitochondrial characteristics including biogenesis and membrane potential have been suggested as key indicators for metabolic fitness and effector function in T cells (163, 189). Enhancing mitochondrial biogenesis through pharmacological or genetic approaches, or by enriching for cells with low mitochondrial membrane potential through cell sorting significantly improves T cell-mediated anti-tumor activities *in vivo* (163, 189–192). Conceivably, nutritional formulations that are tailored to meet the metabolic preferences of cells with different co-stimulatory domains may further enhance the metabolic robustness of CAR T cells.

Immune effector cells have evolved to respond to fluctuations in environmental nutrient levels and thus are able to adapt to environments with either sufficient or insufficient nutrient supply (156). We reason that the pre-adaption of CAR T cells in a conditional media that reproduces the *in vivo* metabolic environment of tumors may improve anti-tumor response *in vivo*. Given that blood flow and oxygen concentration fluctuate in solid tumors, the metabolic stress imposed on CAR T cells may also fluctuate in the TME (193, 194). As such, a fine-tuned adjustment of the severity, duration and frequency of metabolic stress may better recapitulate the metabolic conditions in the TME. Interestingly, transient glutamine restriction *in vitro* via either short-term nutrient starvation or metabolic inhibitor treatment enables a robust antitumor activity of adoptively transferred T effector cells in mouse models of immunotherapy (47, 195–197).

The immune cells response to the changes in the tumor's metabolic microenvironment represents a mechanism of “metabolic checkpoint” that coordinates metabolic status with cellular signaling, and in turn, determines immune function (14). Signaling kinases and transcription factors, such as AMPK and HIF1α can mediate adaptive responses that rewire T cell metabolism to determine immune function. HIF1α-dependent glycolytic pathway is preferentially enhanced in T_H_17 cells than T_reg_ cells. Ablation of HIF1α or pharmacological inhibition of glycolysis reciprocally reduces T_H_17 cells and induce T_reg_ cells differentiation. However, AMPK enhances T_reg_ cells differentiation through negatively regulating OXPHOS (32, 198–205). Intrinsically edited signaling and transcriptional programs may ensure the engagement of fine-tuned metabolic programs to support T cell proliferation (associated with effector functions) or dormancy (associated with memory phenotypes) in response to the changing microenvironment (206). We reason that the engineering of the CAR by integrating a stress response signaling module may ensure a fine-tuned metabolic adaptation of T cells in the TME.

**Figure 3:**
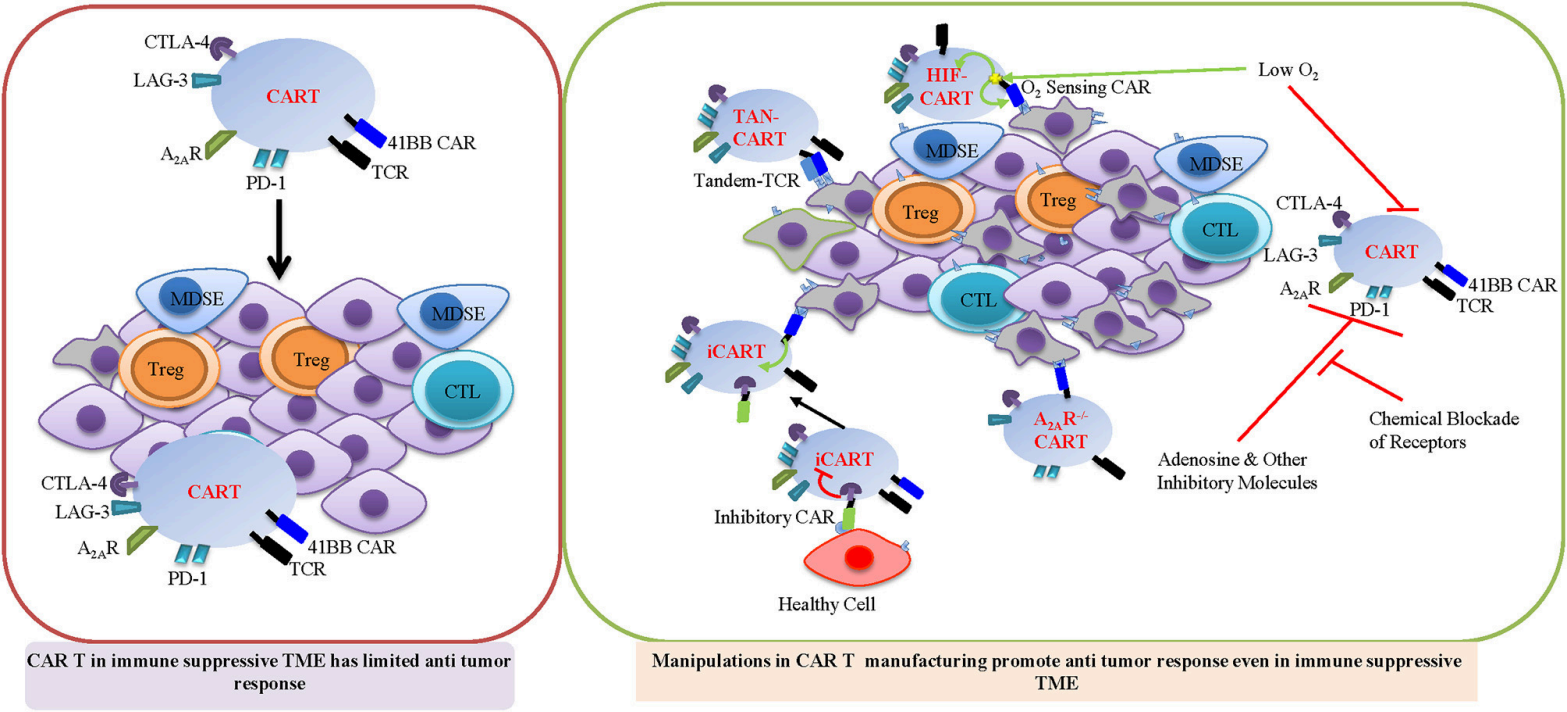
Important strategy for promoting CAR T cell therapy in immune suppressive TME. Manipulations during CAR T manufacturing HIF-CAR, TAN-CAR, and iCAR will promote CAR T function in immune suppressive TME. HIF-CAR T cells are activated by low oxygen concentrations. Inhibitory CAR (iCAR) limits T cell activation in the presence of off-target cells. Tandem CARs (Tan CAR) requires bispecific activation which reduces off target effects. A2AR^−/−^ CAR T cells are resistant to adenosine mediated INF-γ suppression.

The immunosuppressive TME is one of the critical barriers for successful CAR T treatment in the solid tumor. The solid tumor is composed of lymphatic vessels, fibroblasts, infiltrated immune cells, stroma cells, and extracellular matrix (ECM) which constitute the complex TME. During tumor progression and as a result of the heightened glycolytic metabolism of cancer cells, solid tumors are subject to depleted nutrients, acidosis, and hypoxic conditions due to aberrant vascularization. Since hypoxia is a crucial aspect of the TME, targeting hypoxia could be an important strategy for promoting CAR T cell therapy in the solid tumor. Hypoxia induces the stabilization of the HIF-1α transcription factor, which regulates T cell metabolism and function. A recent study designed a novel CAR scaffold that includes the oxygen sensitive domain of HIF1α and provided a proof of concept for engineering microenvironment responsive CAR T cells. T cells which express this engineered HIF1-CAR are cable of responding to a hypoxic environment to display robust cytolytic activity *in vitro* (Figure 3) (207). In addition, CAR T cells that express distinct CAR co-stimulatory domains display different metabolic characters, which in turn impact memory phenotypes. 4-1BBζ CAR T cells exhibit enhanced mitochondrial SRC and mitochondrial biogenesis, which is associated with enhanced central memory phenotypes. Inclusion of CD28ζ in the CAR structure promotes effector memory differentiation and results in increased aerobic glycolysis in CAR T cells (30). In addition to the approach of engineering CAR structure, cytokine formulation can be optimized to modulate metabolic programs and promote the T_scm_ and T_cm_ phenotype that is associated with enhanced CAR T cell persistence and anti-tumor immunity *in vivo* (208). T cells, activated with anti-CD3/CD28 antibody, followed by expansion in the presence of IL-15 and IL-7, not only mimic a more T_scm_ like phenotype, but and also exhibit increased production of IFNγ, TNFα, and IL-2 as well as cytolytic activity against target cells expressing the CAR specific antigen (209, 210). It has been suggested that IL-15 promotes SRC and FAO by upregulating carnitine palmitoyl transferase, a rate-limiting metabolic enzyme that controls FAO. Since T_mem_ cells preferentially engage OXPHOS rather than glycolysis, IL-15 may promote T_mem_ cell differentiation and bioenergetic stability partially by regulating mitochondrial metabolism (162).

### CAR T Cell Preservation

The nutritional optimization of preservative solutions is an important factor for ensuring successful reperfusion and CAR T therapy. As such, a detailed understanding of the metabolic impact on cells during short-/long-term storage or during the reperfusion period is required. The nutritional formulation requires the capability to regenerate ATP, buffer ions and scavenge free radicals in CAR T cells during the period of preservation (211–215). Finally, any inconsistency between the nutrient formulations that are utilized for preservation and those used for *ex vivo* cell manipulation and expansion may potentially render the cells more susceptible to preservation damage.

### CAR T Cell Infusion and Homing to Tumor

Trafficking of T cells into the site of the tumor is required for achieving an optimal CAR T therapeutic response (216, 217). Novel synthetic biology approaches are needed to engineer T cells to logically respond to metabolites that are enriched in the TME, ensuring a fine-tuned tumor recognition and homing response (218, 219). For instance, adenosine is enriched in the TME and suppresses the T cell response partially by engaging cell-surface purinergic receptors-mediated signaling in T cells (220, 221). One theoretical strategy is to engineer an adenosine-responding CAR by fusing the extracellular domain of adenosine receptor to intracellular costimulatory domains, thus artificially switching a normally suppressive signal to an activating signal. We envision that a dual CAR system, with one for a tumor antigen and one for a tumor-associated metabolite, may further enhance the specificity and responsiveness of CAR T cells that may be deployed against tumors in a wide range of sites.

### Tumor Killing *in situ* and Safety Control

The capacity for cell proliferation and persistence in the TME is the best predictor of clinical efficacy in CAR T therapy. Nutritional supplements may overcome the metabolically suppressive microenvironment and may enable CAR T cells to persist and expand, ensuring the optimal “effector vs. target” ratio for the clearance of the tumor *in vivo*. L-arginine is considered a conditionally essential amino acid and supplementation with L-arginine enhances the antitumor response of adoptively transferred T cells in animal models (107). Clearly, arginine is also a metabolic vulnerability of cancer cells in the TME, since circulating arginine is essential for supporting tumor growth (222, 223). Therefore, we need to understand the impact of supplemental nutrients on both T cells and tumor cells to better stratify this approach in the future.

The genetic and enzymatic approaches that enable the conversion of immune suppressive metabolites to either inert compounds or pro-inflammatory compounds are promising strategies to reprogram metabolic TME. The concentration of lactate in vertebrate plasma ranges from 1 to 30 mM under physiological and pathological conditions (224). While lactate accumulated in the TME is generally considered as metabolic “waste,” muscle cells, neurons and certain tumor cells are known to be able to take up and oxidize lactate (225–227). Consistent with these findings, emerging evidence suggests that lactate is a key carbon source *in vivo* and can be oxidized in the mitochondria to generate energy and feed into cataplerotic routes of the TCA cycle (228–236). Enforced expression of phosphoenolpyruvate carboxykinase 1 (PCK1), which presumably increases gluconeogenesis from lactate and thus alleviates the stress from glucose restriction in the TME, has been shown to enhance anti-tumor T cell responses in animal models (167). Also, we envision a rewired lactate flux, by enforcing LDHB expression, may render T cells capable of utilizing tumor-derived lactate, and thus may strengthen the metabolic fitness of CAR T cells in the TME.

One recent study has demonstrated that the systemic delivery of an enzyme that efficiently depletes kynurenine in the TME can enhance the T cell-mediated antitumor immunity (237). Similarly, polyethylene glycol–conjugated adenosine deaminase (PEG-ADA) and ADA gene therapies have been successfully employed to treat ADA-SCID patients (238, 239). Since only immature and transformed T cells display high ADA activity (240–242), systemic ADA treatment through PEG-ADA supplementation may be a new, complementary strategy to optimize the potency and durability of CAR T therapy. It is also conceivable that an engineered oncolytic virus or CAR T cells which express ADA may offer additional strategies to locally deliver ADA into the TME. Nucleoside transporters can rapidly remove adenosine from the extracellular nucleoside pool and direct adenosine into nucleoside salvage to support RNA/DNA synthesis. Thus, expression of high affinity nucleoside transporters may divert adenosine from eliciting an immune-suppressive purinergic signaling response into promoting T cell proliferation (243, 244). Similarly, genetic strategies which enforce the expression and affinity of transports of the key carbon and nitrogen donors (i.e., glucose and glutamine) may confer a selective advantage on T cells over tumor cells in the TME.

Another promising therapeutic strategy for remodeling the microenvironment is to modulate ion and pH balances. Reducing the potassium level through enforced expression of potassium channels in T cells can enhance their antitumor activity (122). A lactate-binding compound has been recently developed as a potent pharmaceutical approach for engineering metabolic flux of lactate and normalizing the pH *in vivo* (245).

The naturally occurring T cell mediated response is largely controlled through cell autonomous mechanisms. However, CAR T therapies often lead to a series of adverse effects that may be reduced through the fine-tuned regulation of the timing, location and amplitude of T cell activity. The principal of the recently developed “ON-switch” CARs can be theoretically extended by employing “metabolic switches” to control T cell activities in real-time (246, 247). In addition, the engineering of metabolic enzymes to activate prodrugs has been exploited as a suicide gene system (248–250). A similar strategy can be exploited as a “safety switch” to enable the selective ablation of CAR T cells at will by providing prodrugs and therefore limiting the on-target, but off-tumor toxicities of CAR T cells (251–253).

## Future Directions

Cancer cachexia, which is responsible for the death of more than 20% of cancer patients, is characterized by dramatic body weight loss and disproportionate wasting of skeletal muscle (254–256). Supportive care including dietary treatment and physical exercise that can maintain energy balance, as well as increase insulin sensitivity, protein synthesis rate, and anti-oxidative enzyme activity, which is beneficial for relieving symptoms of cachexia (257–260). Similar strategies may be applied as systemic approaches to enhance anti-tumor immunity by fostering an optimized metabolic environment for immune cells.

The fast moving CAR T therapy field has generated tremendous excitement and will likely change the paradigm of therapeutic interventions for solid tumors. Manipulation of metabolism has been demonstrated as a potential approach to the regulation of immune responses in various preclinical settings. Beyond CAR T therapy, the above potential metabolic modulations can also be considered as adjunctive therapy together with other immunotherapies, including checkpoint blockade and cancer vaccines (261–265). TME consists of an intricate, highly complex and dynamic network of immune cell subsets. Other immune cell subsets, such as natural killer (NK) cells, TAM and MDSC are universally found in the TME and are key players in anti-tumor immunity (266, 267). We will need to continue to decipher the essential metabolic pathways by analyzing metabolic flux and assessing the consequences of metabolic intervention on these pathways in all key cellular components of the TME. Understanding how control of (or by) metabolic pathways impacts anti-tumor immune responses in these cell types is required to selectively strengthen the metabolic fitness in effector T cells and potentiate the metabolic vulnerabilities of tumor cells and immune-suppressive cells. Finally, Strategies to improve immune cell metabolic fitness may be applicable across a broad panel of cancer immunotherapies including checkpoint blockade and cancer vaccines (261–265).

## Author Contributions

XX, JG, and JS wrote the manuscript and RW wrote and finalize the manuscript.

### Conflict of Interest Statement

The authors declare that the research was conducted in the absence of any commercial or financial relationships that could be construed as a potential conflict of interest. The handling editor declared a shared affiliation, though no other collaboration, with the authors at the time of review.
